# Metals in ALS TDP-43 Pathology

**DOI:** 10.3390/ijms222212193

**Published:** 2021-11-11

**Authors:** Lassi Koski, Cecilia Ronnevi, Elina Berntsson, Sebastian K. T. S. Wärmländer, Per M. Roos

**Affiliations:** 1 Institute of Environmental Medicine, Karolinska Institutet, 171 77 Stockholm, Sweden; per.roos@ki.se; 2 Capio St. Göran Hospital, 112 19 Stockholm, Sweden; ceciliaronnevi@gmail.com; 3 Department of Biochemistry and Biophysics, Stockholm University, 106 91 Stockholm, Sweden; elina.berntsson@dbb.su.se; 4 Department of Chemistry and Biotechnology, Tallinn University of Technology, 12616 Tallinn, Estonia

**Keywords:** neurodegeneration, proteinopathy, metallopathy, protein aggregation, amyloid, metal exposure, metal–protein binding

## Abstract

Amyotrophic lateral sclerosis (ALS), Alzheimer’s disease, Parkinson’s disease and similar neurodegenerative disorders take their toll on patients, caregivers and society. A common denominator for these disorders is the accumulation of aggregated proteins in nerve cells, yet the triggers for these aggregation processes are currently unknown. In ALS, protein aggregation has been described for the SOD1, C9orf72, FUS and TDP-43 proteins. The latter is a nuclear protein normally binding to both DNA and RNA, contributing to gene expression and mRNA life cycle regulation. TDP-43 seems to have a specific role in ALS pathogenesis, and ubiquitinated and hyperphosphorylated cytoplasmic inclusions of aggregated TDP-43 are present in nerve cells in almost all sporadic ALS cases. ALS pathology appears to include metal imbalances, and environmental metal exposure is a known risk factor in ALS. However, studies on metal-to-TDP-43 interactions are scarce, even though this protein seems to have the capacity to bind to metals. This review discusses the possible role of metals in TDP-43 aggregation, with respect to ALS pathology.

## 1. Introduction

Amyotrophic lateral sclerosis (ALS) is a fatal neurodegenerative disease characterised by a specific and progressive demise of upper and lower motor neurons in the motor cortex, brainstem and spinal cord [1,2,3]. Death occurs within 3 to 5 years from onset due to respiratory failure caused by paralysis and wasting of voluntary muscles [2,4,5]. Even though multiple environmental risk factors have been recognised, ranging from smoking and childhood infections to metal exposure and high-performance sport and military service [1,6], the mechanisms of pathogenesis in ALS remain elusive. To date, effective treatment of ALS is lacking [7]. An important aspect of the disease appears to be protein alterations and accumulation of protein aggregates, occurring intracellularly and extracellularly, in tissues, blood and to some extent also in cerebrospinal fluid (CSF) [5,7,8,9].

**Figure 1:**
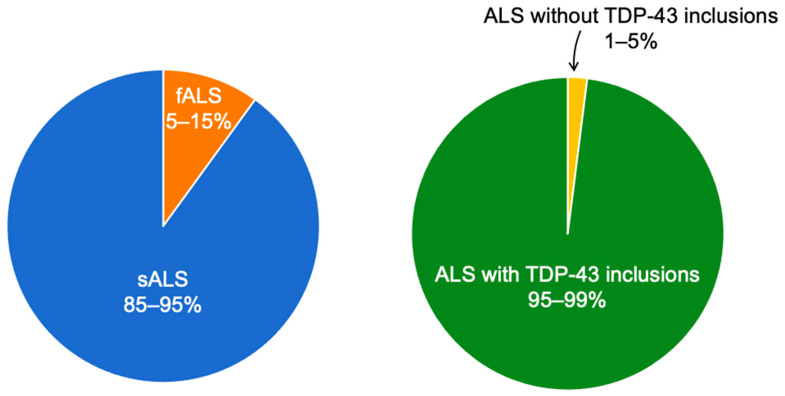
Left: ALS is classified into sporadic (sALS) and familial (fALS) cases [1]. Right: inclusions of the TDP-43 protein are found in the majority of ALS cases [9]. Original drawing by L.K.

In the United States of America and Europe, the ALS incidence is approximately 1–2 per 100,000 person-years, the prevalence is 3–5 per 100,000 persons, and the cumulative lifetime risk of ALS is approximately 1 in 400 [2]. About 5–15% of cases are familial (fALS), mainly inherited as dominant traits. The remaining 85–95% of ALS cases are sporadic (sALS) with no family history of the disease and with a suspected environmental aetiology (Figure 1) [1]. Some 20% of ALS cases, mostly bearing the *C9orf72* hexanucleotide repeat extension, develop frontotemporal lobar degeneration (FTLD) and progressive cognitive decline with behavioural changes subsequently leading to clinical frontotemporal dementia (FTD) [2]. This makes it likely that ALS and FTD are variants within a spectrum disorder [3].

ALS patients and controls differ in the abundance of inflammatory marker proteins [10], and a few CSF proteins involved in complement activation and the acute phase response have shown possible prognostic potential for ALS [11]. However, no major differences in CSF protein composition in ALS vs. controls have been established [12], and the idea of creating an ALS biomarker profile from CSF proteins has not gained momentum [13]. In peripheral tissues, the situation is different, and several studies have reported significant amounts of aggregates [14] of proteins such as SOD1 [15,16,17], C9orf72 [17,18,19], FUS [15,20], and TDP-43 [15,17,20,21] in ALS blood and tissues. This difference might not be surprising, given the efficient barriers between the CSF compartment and the peripheral blood/tissues, which protect the central nervous system from environmental influences [22,23]. In a situation where protein aberrations are induced by external influences—a likely scenario in sALS—more protein alterations may be expected in peripheral blood and tissues than in the CSF [11,24].

In 1993, *SOD1* was identified as the first gene associated with ALS [1,25]; since then, numerous other related genes have been discovered, including *TARDBP*, *C9orf72* and *FUS* [26,27]. Many of the discovered ALS genes encode proteins that interact with nucleic acids. The first such protein to be discovered was a 43 kDa transactive response DNA-binding protein known as TDP-43, encoded by the *TARDBP* gene on chromosome 1 and binding to pyrimidine-rich transactivation response (TAR) regions [2,5,7,28,29]. TDP-43 normally exists in the cell nucleus, and it is abundantly expressed in all tissues including the brain [28]. It was originally identified as a repressor of the transcription of human immunodeficiency virus type 1-gene, but later research has established a wide range of functions. TDP-43 is now considered to be involved in many cellular processes and specifically in gene expression, by binding to DNA and RNA, and by regulating messenger ribonucleic acid (mRNA) splicing, translation, transportation, and degradation [29,30].

Dislocation of TDP-43 from the nucleus into the cytosol and formation of ubiquitinated and hyperphosphorylated cytoplasmic inclusions of aggregated TDP-43 [31,32] have been detected in all sALS cases, in *SOD1* mutation-negative fALS and in FTLD (Figure 1) [9]. An absence of TDP-43 immunoreactivity has been consistently reported in mutant *SOD1* transgenic mice [5]. Later, it became known that mutations in the *TARDBP* gene may induce fALS [33]. Full-length TDP-43 proteins tend to aggregate in the spinal cord, whereas aggregates of C-terminal TDP-43 fragments (CTFs) appear almost exclusively in the brain and not in the spinal cord [34]. TDP-43 also forms pathognomonic aggregates in limbic-predominant age-related TDP-43 encephalopathy (LATE), where the TDP-43 morphology is identical with the pattern seen in ALS [35,36]. LATE clinically mimics Alzheimer’s disease (AD), with some researchers implying that a substantial portion of clinical AD diagnoses could in fact be attributed to LATE [35,36].

The relationship between the inclusions of aggregated TDP-43 and ALS pathology and progression remains unclear and various mechanisms have been proposed [1,5,7,28,34,37,38]. The fundamental problem has been argued to lie in an imbalance between nuclear and cytoplasmic TDP-43 [39], yet such hypotheses do not explain the origin of this imbalance. Mutations in the *TARDBP* gene have been reported in approximately 3% of fALS and in 1.5% of sALS cases [1], possibly explaining TDP-43 aggregation in patients with these mutations. Otherwise, TDP-43 inclusions have been shown to be formed secondary to mitochondrial damage, mediated by caspase and calpain activation [40]. Oxidative stress has been described as a possible cause of TDP-43 aggregation, as inducers of oxidative stress have been demonstrated to delocalise the protein into the cytoplasm, where it then forms aggregates [41]. Interestingly, oxidative stress is known to alter pre-mRNA splicing patterns regulated by TDP-43 [41]. Furthermore, it has been suggested that the presence of cytoplasmic and intranuclear TDP-43 inclusions may exert neuronal toxicity via a gain of function, or via disruption of physiological functions of TDP-43 in the nucleus, from which it is depleted [42].

Aggregation of proteins into toxic species is a hallmark of many diseases known as proteinopathies, including the amyloid-β (Aβ) peptides and tau proteins in AD [43], the α-synuclein (α-Syn) protein in Parkinson’s disease [44,45], the prion (PrP) protein in Creutzfeldt–Jakob disease [46,47] and the huntingtin protein in Huntington’s disease [48]. In vivo, the protein aggregates can be detected after staining with a dye or other biomarker. In vitro, the protein aggregates contain regular fibrillar structures that can be visualised by techniques such as electron microscopy (EM) or atomic force microscopy (AFM) [49,50]. It is also possible to characterise the size and charge of the aggregates [51] as well as their aggregation rate, kinetics, formation mechanism and early oligomeric states [52,53].

A common structural motif in the fibrillar aggregates is the “cross-β structure”, which consists of repeated units of β-strands positioned perpendicular to the fibril axis, stabilised by inter-strand hydrogen bonds aligned along the direction of the fibril axis [54,55]. The main toxic species in many proteinopathies appears not to be the large protein aggregates but rather small soluble intermediate oligomers that form along the aggregation pathways [44,46,48,56]. Being important potential drug targets, these oligomers have been extensively researched, but their sizes, shapes and structures remain unclear [49]. The α-pleated sheet structure might be an important conformational motif of aggregates [57]. The oligomers may spread from cell to cell via, e.g., exosomes [48,56], and a possible toxic mechanism for these oligomers is the disruption of cell membranes [49,58]. The formation of oligomers and other protein aggregates can be influenced by a number of factors, including other proteins, small molecules and metal ions [58,59,60,61,62,63]. 

In ALS studies, astrocyte activation by manganese (Mn) and lead (Pb) was found to promote intracellular protein aggregation [64], and elevated metal ion levels were observed in a TDP-43^A315T^ transgenic mouse ALS model [65]. In a *SOD1^G93A^* mouse ALS model, the copper (Cu) homeostasis was disturbed, and Cu accumulated in the spinal cord of the mice [66]. Interestingly, the administration of a Cu-chelating agent (ammonium tetrathiomolybdate) enhanced survival and slowed ALS progression, even when the chelator therapy was introduced after disease onset [66]. Among the various environmental influences possibly contributing to ALS [6,67], neurotoxic metals [68] stand out as being tentatively involved in the common protein dysregulations and aggregations observed in ALS peripheral blood and tissues. This review discusses the role of metals in TDP-43 aggregation and their relevance in ALS pathogenesis.

## 2. ALS and Metal Exposure

Exposure to metals has for a long time been hypothesised to contribute to sALS [6,69,70,71] in ways that seem to be varied, individual and complex [71]. Occupational exposures associated with higher risk of ALS are found in precision-tool manufacturing workers (odds ratio [OR]: 1.68; 95% confidence interval [95% CI]: 1.11, 2.52) and in glass, pottery and tile workers (OR: 1.76; 95% CI: 1.03, 3.00) [72]. Both of these occupations involve a known risk for exposure to metals with neurotoxic properties [73,74]. Case reports describing occupational metal exposure preceding ALS exist, as well as other specific and varied circumstances where exposure to metals with neurotoxic properties have preceded ALS onset. Thus, a 44-year-old ALS patient died after 9 years of heavy cadmium (Cd) exposure in a nickel-cadmium battery factory [75]. An Algerian woman was diagnosed with ALS after repeated respiratory exposure to Pb fumes from melting Pb [76]. A Korean electronic parts manufacturing worker exposed to Pb [77] and other metals, with a blood Pb concentration of 31 μg/dL half a year after ceased exposure, developed fasciculations, weakness and muscle atrophy diagnosed as ALS. A man in Kenya developed ALS after severe exposure to potassium permanganate (KMnO_4_) as part of a traditional African medicine procedure [78]. Of special interest is an old report of bulbar ALS following Mn intoxication [79]. Other case reports describing ALS following various Mn exposures such as Mn mining or welding have been published (summarised in [80]). Several other case reports with suspected or verified exposure to other neurotoxic metals such as mercury (Hg) and aluminium (Al) preceding ALS symptoms can also be extracted [81,82,83,84]. In systematic studies, similar connections between exposure to metals and ALS onset can be seen. Occupational exposure to Pb was significantly (OR = 5.7) more common in ALS patients than in controls in a Scottish study [85]. In a series of 31 ALS cases, it was found that 24 out of those 31 had a history of metal exposure preceding initial symptoms of ALS [86]. Other studies also report associations between metal exposure and ALS [87,88]. Interestingly, in 2017, a group of German medical researchers claimed complete remission in an ALS patient after having treated him for Hg intoxication [89]. Thus, several disparate metal exposure situations seem to be associated with sALS.

In addition to reports of environmental metal exposure preceding clinical ALS, elevated metal concentrations have been measured in body fluids and tissues from ALS patients. Elevated plasma Pb concentrations have been reported in ALS [90,91]; however, reports on metal blood concentrations have often been inconsistent [69]. In the CSF of ALS patients, concentrations of several other metal ions, these being Al, Cd, cobalt (Co), Cu, Mn, Pb, uranium (U), vanadium (V) and zinc (Zn), have been shown to be significantly elevated [68]. Elevated CSF concentrations of selenium (Se) in the form of selenite ions (SeO_3_^2−^), possibly secondary to exposure via drinking water, have been linked to endemic clusters of ALS [6,92]. Iron (Fe) has been observed to accumulate in the brain, spinal cord and CSF of ALS patients [93], who also display elevated blood ferritin concentrations [41,93].

The pathophysiological mechanisms of ALS have been broadly studied. Glutamate-induced excitotoxicity has been used to support the so-called “dying-forward” hypothesis, according to which the neurodegeneration is initiated in the upper motor neurons [1]. This hypothesised anterograde progression of the disease is explained by excessive activation of glutamate receptors, known to induce intracellular calcium ion dyshomeostasis, reactive oxygen species (ROS) and mitochondrial dysfunction, leading to cell death [1,40,94]. The opposing “dying-back” hypothesis presumes that the disease originates at the neuromuscular junctions of the lower motor neurons, progressing retrogradely to upper motor neurons [1,40]. This hypothesis is consistent with the idea of retrograde axonal transport of metals from muscles and/or the periphery into the central nervous system [95,96]. Some researchers have even suggested independent parallel processes at these two motor neuron levels [1,40]. Cu-Zn-superoxide dismutase (SOD1), coded for by the *SOD1* gene, normally protects cells against ROS [41], and approximately 10–20% of fALS and 1–5% of sALS is known to be associated with a mutation in *SOD1* [6]. The role of ROS in both *SOD1* mutations [6] and glutamate-induced excitotoxicity [94] has led to theories of the possible role of oxidative stress in ALS [41]. Impaired metal homeostasis has also been suggested as a possible mechanism, as, e.g., Fe and Cu ions participate in cellular redox reactions and can produce harmful oxygen radicals [40,41]. Both metals also act as cofactors in mitochondrial enzymes [6], the activity of which has recently been shown to be reduced in ALS patients [40].

When discussing metal exposure in ALS and possible metal-related pathophysiological mechanisms in ALS, a distinction between metals without any biological function and biologically relevant metals must be made. Metals such as Pb, Hg, Cd, Al, V and U are alien to cellular metabolism, toxic in trace amounts and have no defined role in biological systems, in contrast to metals and metalloids such as Mn, Zn, Cu, Fe, Co and Se, which, in physiological concentrations, are vital to protein function and are constitutional parts of many metal-specific proteins such as ceruloplasmin and haemoglobin [97]. The latter category typically shows a U-shaped curve of toxicity vs. concentration with a harmful deficiency with symptoms, an optimum for biological function and toxicity at higher concentrations [98]. The former category shows toxicity only, and a meaningful lower limit for safe exposure is hard to define [97]. Furthermore, a metallic or metalloid element may present itself in different chemical states: as an uncharged solid metal, as charged ions of different valency such as Se^2−^ and Se^4+^, or as part of a molecule or a polyatomic ion such as SeO_3_^2−^. Although many experimental methods only detect the amount of the element as such in, e.g., a blood or tissue sample, the different chemical states have different properties and may interact differently with biological compounds. For example, Pb^4+^ ions display residue-specific and relatively strong binding to the Aβ peptide involved in AD, while Pb^2+^ ions do not [60].

## 3. The TDP-43 Protein and Its Aggregation

**Figure 2:**
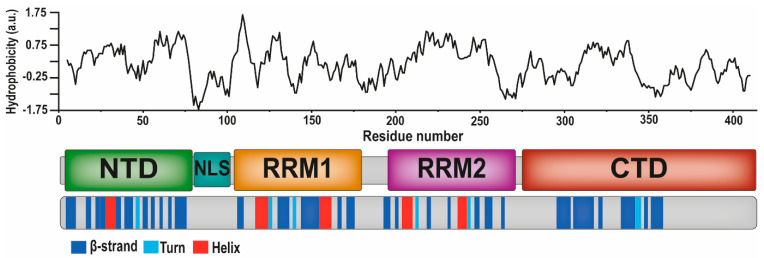
The different domains of the TDP-43 protein, including their hydrophobicities and secondary structures (β-strand, turn, helix, or unstructured). NTD—N-terminal domain; NLS—nuclear localization signal domain; RRM1 and RRM2—RNA recognition motifs 1 and 2; CTD—C-terminal domain [34,101]. Original drawing by E.B.

TDP-43 is a heterogeneous nuclear ribonucleoprotein consisting of 414 amino acids, thought to be natively dimeric or to occur in a monomer-dimer equilibrium [99,100]. Figure 2 presents the primary sequence of the TDP-43 protein in a schematic way, showing the structured regions, the five most important domains, and the calculated hydrophobicity of the amino acid sequence. The structural information is based on the review articles by, respectively, Berning and Walker [34] and François-Moutal et al. [101], together with the information about the TDP-43 protein in the UniProt database (ref. number Q13148). The hydrophobicity values were calculated using the ProtScale software [102] and parameters provided by Abraham and Leo, 1987 [103]. The C-terminal domain (Figure 2) is glycine-rich and mainly disordered [104], and several ALS-related mutations are located there [100]. The N-terminal domain (Figure 2) contains six beta-strands and one alpha helix [105] and is followed by two tandem RNA recognition motifs—RRM1 and RRM2, collectively referred to as RRM1/2—that are highly conserved and important for binding nucleic acids [28,106]. The RRMs display the so-called RRM fold, where four anti-parallel beta-strands alternate with two α-helices (β1α1β2β3α2β4) and where two additional beta-hairpins increase the surface for RNA binding [107]. A nuclear localisation signal (NLS) domain is positioned between the N-terminal and RRM1 domains (Figure 2). Mutations in the NLS sequence have been shown to impede recognition by the proteins that transport TDP-43 into the nucleus [39].

Many studies have been conducted on TDP-43 fragments and fewer on the full-length protein. Some segments in the C-terminal domain have not been fully characterised structurally or otherwise, and they are likely unstructured. As inclusions from the cytoplasm of ALS and FTLD patients often contain a mixture of fragments and full-length protein [32], studying fragments is motivated, but understanding the properties of full-length TDP-43 may require more than understanding the properties of the parts. The protein contains several hydrophobic regions (Figure 2), suggesting that aggregation could be initiated at many places. Both in vitro and in-cell studies have shown that the N-terminal domain can aggregate into dimers and oligomers [108,109]. Some studies suggest that the RRM1 and RRM2 domains can also aggregate on their own after misfolding and thus contribute to TDP-43 proteinopathy by nucleation, propagation or by providing an aggregation pathway for the C-terminal domain [110]. One study reported that the C-terminal domain containing amino acid residues 311–320 was critical for CTF aggregation and that the residue 246–255 region promoted CTF aggregation even though it did not nucleate on its own [111]. Several other studies also indicate that the C-terminus of the TDP-43 protein contains various regions important for aggregation [112,113].

TDP-43 is normally located in the neuronal nucleus, and it is continually shuttled between the nucleus and the cytoplasm. It contributes to the repression of gene expression, pre-mRNA splicing and autoregulation of its own nuclear mRNA. In the large majority of ALS and FTLD cases, pathological TDP-43 is phosphorylated, ubiquitinated, and aggregated into insoluble cytoplasmic inclusions, where TDP-43 is the major inclusion component [5,7,42]. Simultaneously, ALS patients present decreased amounts of TDP-43 protein in the nucleus. The accumulation of TDP-43 in the cytoplasm might be enhanced by cellular energy deficits, and the imbalance between nuclear and cytoplasmatic TDP-43 per se might play a role in ALS pathophysiology [40]. Nuclear egress of TDP-43 is size-dependent and driven mainly by passive diffusion in the absence of a nuclear export signal. Simultaneously, cytoplasmic TDP-43 is translocated into the nucleus by the classical pathway driven by a gradient of Ras-related nuclear GTPase-activating protein. This makes it likely that while cellular energy deficits have no effect on the nuclear export of TDP-43, it can impair its nuclear import from the cytoplasm, resulting in abnormal accumulation of TDP-43 in the cytoplasm [40].

The idea that metal ions could interact with the TDP-43 protein and affect its aggregation and toxicity has gained increasing interest in recent years. Below, we present the current evidence for potential interactions between TDP-43 and certain metal ions and their possible effects on the harmful TDP-43 aggregation typically seen in ALS pathology.

## 4. Lead and TDP-43

Pb has been suspected to be a factor contributing to neurodegenerative disease for decades, and it has been thoroughly studied for its neurotoxic properties [97]. Several studies have demonstrated significantly elevated blood Pb concentrations in ALS patients [90,91], and Pb has also been found to be elevated in ALS CFS [68]. A large majority of absorbed Pb is stored in skeletal tissue, reflecting lifetime Pb exposure [97].

In a recent study by Ash et al. [42], a total of 91 potential toxicants were screened for their ability to stimulate TDP-43 aggregation. In this screening, lead (II) acetate trihydrate was found to significantly increase nuclear TDP-43 aggregates in rat adrenal medulla cells without significantly increasing cell death. In further experiments, treatment of murine hippocampal cells with lead (II) acetate trihydrate induced, in a biphasic manner, TDP-43 inclusions in the nucleus [42], as opposed to the cytoplasmic TDP-43 inclusions typically seen in ALS [31,32]. In addition, lead (II) acetate trihydrate increased the TDP-43-mediated mRNA splicing in primary cortical neurons and induced accumulation of insoluble TDP-43 in rat adrenal medulla cells [42]. This study provided the first ever evidence indicating a possible role for Pb^2+^ ions in TDP-43 aggregation in vitro.

The idea that Pb could induce TDP-43 pathology and thus contribute to the development of LATE was presented for the first time in 2020 by Fuller-Thomson et al. [36]. They proposed a possible design for a future cohort study, with bone samples to measure environmental Pb exposure and brain autopsies to confirm LATE [36]. However, such studies have yet to be carried out.

## 5. Mercury and TDP-43

Hg exists in nature in organic and inorganic forms, both of which are known to be toxic to organisms [97]. Organic Hg, such as methyl mercury (MeHg) and ethyl mercury (EtHg), is generally considered more toxic than its inorganic forms, including elemental Hg and mercuric salts [97]. Ingested, injected or inhaled Hg accumulate in anterior horn cells of the spinal cord but not in surrounding spinal cord tissue after Hg exposure of primates [114] and rodents [71,115,116,117,118]. A few case reports have linked Hg exposure to ALS [81,83,89,119,120,121,122,123].

Due to these features, Hg was one of the first metals studied in relation to a possible effect on TDP-43 aggregation. In 2011, Pamphlett et al. [124] exposed mice to different inorganic compounds containing Hg. Spinal cord tissue blocks were stained for Hg and simultaneously immunostained for TDP-43. While Hg was seen to accumulate in the lateral segments of the spinal cords, no TDP-43 inclusions characteristic of ALS were detected in the exposed mice. Additionally, no objective signs of motor neuron disease were observed in the mice. The difficulties in measuring tissue Hg should be taken into consideration when interpreting these results [125].

In contrast, the 2019 screening of 91 potential environmental toxicants by Ash et al. [42] demonstrated that, in addition to Pb, MeHg was able to increase nuclear TDP-43 aggregates and, unlike Pb, increase cell death [42]. Methylmercuric (II) chloride was then shown to induce nuclear TDP-43 inclusions in a dose-dependent manner in murine hippocampal cells. Systemic administration of MeHg via drinking water to mice also induced TDP-43 aggregates as well as TDP-43-mediated mRNA splicing in cortical tissue [42]. These findings suggest that MeHg could have a pivotal role in TDP-43 aggregation.

## 6. Zinc and TDP-43

Zn is an essential metal which becomes toxic in excessive amounts [97]. Zn has been recognised to possibly contribute to AD [126], and, in recent years, its binding to Aβ, the protein that typically aggregates in AD, has been thoroughly studied [126,127]. Zn has been found to be elevated in ALS CSF [68], as well as in the spinal cord of transgenic mice with mutant TDP-43 causing locomotor impairment [65].

Due to the abundant previous research on the effects of Zn on several neurodegenerative diseases, it was no surprise that Zn was one of the first metals to be studied in relation to TDP-43 aggregation. In 2010, Caragounis et al. [62] reported that overnight treatment of neuronal-like cells (SY5Y) with ZnCl_2_ significantly decreased their TDP-43 level and induced aggregation of both nuclear and cytoplasmic TDP-43, significantly decreasing the viability of these cells. These inclusions were found to consist of full-length, non-phosphorylated, non-ubiquitinated TDP-43 [62], as opposed to the hyperphosphorylated and ubiquitinated, sometimes fragmented peptides typically seen in ALS [31,32]. Additional experiments suggested that the toxic effects could be ROS-mediated, whereas depletion of nitric oxide (NO) did not inhibit the observed Zn-mediated aggregation [62], suggesting the mechanism to be independent of NO. Hence, Zn was the first metal demonstrated to induce TDP-43 aggregation in vitro.

Zn is so far also the only metal for which the binding to TDP-43 has been characterised. In a study by Garnier et al. [128], the RRM1/2 region of TDP-43 was analysed by electrospray ionisation mass spectrometry, and Zn was observed to bind to RRM12 at two different binding sites, corresponding to the species [RRM1/2 + 2 Zn^2+^ + 5 H^+^]^9+^ with an approximate mass of 20,098 Da [128]. In further experiments designed to assess if Zn affects the tertiary structure of RRM1/2, Zn binding was demonstrated to decrease the thermostability of RRM1/2, causing denaturation at temperatures as low as 40 °C. Subsequent dynamic light scattering experiments revealed that while RRM1/2 was shown to exist in monomers in the absence of Zn, the presence of Zn was observed to induce the formation of oligomeric aggregates of the protein, which EM later revealed to be two different species, one larger and one much smaller [128]. Simultaneously, the monomeric species were shown to completely disappear [128]. The oligomers were later demonstrated to cause a significant increase in the fluorescence of thioflavin T (ThT), a typical marker for amyloid aggregates [129]; furthermore, three possible aggregation-prone segments could be detected within the RRM1/2 sequence [128]. Later, a Zn-binding site in the RRM2 domain has been described, with coordinating amino acids likely involving the negatively charged glutamate E261 and the two histidines H256 and H264 [130].

## 7. Other Metals and TDP-43

Fe and Cu have important roles in intracellular redox reactions [40,41,131,132]. Concentrations of both Fe and Cu have been shown to be elevated in ALS CSF [68,93], and Fe has additionally been shown to accumulate in the central nervous system of ALS patients [93]. However, Caragounis et al. [62] were unable to detect a decreased TDP-43 level or increased aggregation in neuronal-like SY5Y cells treated with Cu and Fe salts, although these changes were observed after exposure to a Zn salt. Parker et al. [133] later demonstrated that the treatment of SY5Y cells with copper (II) *bis*(thiosemicarbazonato complexes inhibited TDP-43 aggregation, likely via the inhibition of phosphorylation, suggesting a neuroprotective function of the compound.

In a recent case report study, Mitani et al. [134] described a Japanese male ALS patient presenting with speech apraxia and upper motor neuron symptoms, combined with post-mortem histopathology of TDP-43 aggregation and Fe accumulation in the prefrontal gyrus and the frontal operculum. While these atypical symptoms corresponded with the pathological changes in relevant brain regions, it remained unclear to what extent the TDP-43 aggregation and the Fe accumulation were causatively associated [134].

Elevated Mn concentrations have been observed in CSF from ALS patients [135] as well as in the spinal cord of TDP-43-transgenic mice with locomotor impairment [65]. Two Mn-containing compounds, manganese acetate and methylcyclopentadienyl manganese tricarbonyl, were included in the screening by Ash et al. [42], but these did not cause any significant change in TDP-43 homeostasis or cell viability.

## 8. Metal-Induced TDP-43 Aggregation: A Possible Pathological Mechanism in ALS?

Aggregation of TDP-43 has been observed in the majority of all studied ALS cases (Figure 1) [9,31,32]. Protein aggregation is influenced by several physico-chemical variables, such as changes in pH and temperature [59], interactions with other proteins [63,136,137,138,139], and by binding of metal ions to specific metal-binding regions [52,60,61,62,125,140,141]. Six metals with known neurotoxic properties have been studied in relation to TDP-43 so far, i.e., Pb, Hg, Zn, Fe, Cu and Mn (Table 1). Of these metals, Pb, MeHg [42] and Zn [62,128] have been reported to induce the aggregation of TDP-43 in vitro, whereas similar experiments with Fe, Cu [62] and Mn [42] have failed to show any effect on TDP-43 aggregation. Focal Fe accumulation has been linked to TDP-43 aggregation in atypical ALS [134], while Cu-containing compounds have rather been reported to prevent TDP-43 accumulation [133]. MeHg has been observed to induce TDP-43 aggregation also in vivo [42], while inorganic Hg has been shown not to [124]. It appears that the chemical form of Hg affects its ability to influence TDP-43 aggregation, a conclusion supported by organic Hg being previously known to be more toxic than inorganic Hg [97].

Zn is the only metal where binding to TDP-43 has been characterised at the molecular level [128]. RRM1/2 was shown to bind a total of two Zn ions, which induced oligomerisation and aggregation of the protein [128]. Later, a Zn-binding site in the RRM2 domain has been described, likely involving the negatively charged glutamate E261 and the two histidines H256 and H264 [130]. Similar results have been observed for the binding of Zn ions to histidine residues in PrP [141,142]. However, binding of a single Zn ion to the Aβ peptide involved in AD, mainly via histidines, promoted disordered aggregation and retarded formation of regular amyloid fibrils [61,143,144]. ThT and thioflavin S (ThS) fluorescence has been shown to significantly increase upon binding to amyloid aggregates [129]. Similar patterns have been observed also in experiments with a spinal cord subset of full-length TDP-43 aggregates and ThS [145], and with Zn-induced RRM1/2 aggregates and ThT [128]. Aggregation-prone hydrophobic segments in the amyloid sequence have been identified by different methods to predict amyloidicity, and three similar regions have been detected in RRM1/2 using these methods [128]. Given these shared characteristics of the Zn-induced RRM1/2 aggregates and the amyloid aggregates seen in AD, it would be reasonable to argue that these RRM1/2 aggregates might have some pathogenic properties. However, aggregates involving RRM1/2 fragments have not been reported in vivo in ALS patients.

Full-length TDP-43 aggregates are predominantly detected in the spinal cord [34], and some of the aggregates inside the spinal cord display similar amyloid characteristics as RRM1/2 aggregates [128,145]. Aggregates consisting of CTFs are mostly found in the cerebral cortex [34], where aggregates do not display the same amyloid characteristics as RRM1/2 aggregates [128,145]. The likely similarities between RRM1/2 and full-length TDP-43 aggregates suggest the importance of the RRM1/2 region for full-length protein aggregation. One possible mechanism for Zn ions inducing aggregation of the endogenous full-length protein [62], as well as of RRM1/2 fragments [128,130], could be Zn binding to RRM1/2 residues from two different TDP-43 molecules/fragments, thereby bringing the two together and inducing aggregation.

In this review, six metals have been discussed in relation to TDP-43, yet several other metals may correlate with ALS and/or other TDP-43 proteinopathies, such as Cd and Al (Table 1). Applying previously described in vitro approaches [42,62] to investigate various neurotoxic metals could help elucidate whether other metal ions can induce TDP-43 aggregation in these diseases. 

## 9. Future Perspectives

The roles of metal imbalances and metal exposure in neurodegenerative disorders remain to be clarified. Many of these disorders have been proposed to be metallopathies [146], as metal imbalances are involved in their pathologies. MeHg, Pb and Zn contribute to TDP-43 aggregation (Table 1), and TDP-43 aggregates are found in the cytoplasm of nerve cells in almost all sALS cases (Figure 1). Further studies should investigate, at the molecular level, the binding properties of different metal ions to the TDP-43 protein (and to other proteins known to aggregate in ALS), and establish dissociation constants, binding ligands, order of competition at metal binding sites, and effects on protein aggregation and toxicity. The implementation of such methods into clinical ALS research may help in developing methods for the diagnosis and treatment of ALS.

## 10. Conclusions

Metal imbalance and/or environmental exposure to neurotoxic metals may contribute to the harmful aggregation of the protein TDP-43 as seen in amyotrophic lateral sclerosis.

## Figures and Tables

**Table 1:** Metals in cerebrospinal fluid (CSF) of ALS patients, and metals studied in relation to TDP-43 aggregation. In column 2, metals found to be significantly elevated (↑) or unchanged (–) in ALS CSF [68,71] are indicated. Columns 3 and 4 show metals demonstrated to induce (↑) or not to induce (–) aggregation of TDP-43 and the studies where these were studied, respectively [42,62,124,128]. Lead (Pb) and zinc (Zn) have both been shown to induce TDP-43 aggregation and to be significantly elevated in ALS CSF. Methyl mercury (MeHg) has not been studied in ALS CSF.

Metal	CSF	TDP-43	Study Model	Ref.
**Al**	↑			[68,71]
**Cd**	↑			[68,71]
**Co**	↑			[68,71]
**Cu**	↑	–	In vitro	[62,68,71]
**Fe**	–	–	In vitro	[62,68,71]
**Hg**	–	–	In vivo	[68,71,124]
**MeHg**		↑	In vitro, In vivo	[42,68,71]
**Mn**	↑	–	In vitro	[42,68,71]
**Pb**	↑	↑	In vitro	[42,68,71]
**U**	↑			[68,71]
**V**	↑			[68,71]
**Zn**	↑	↑	In vitro	[62,68,71]
		↑	In vitro	[68,71,128]
